# Sun Stroke

**Published:** 1885-09

**Authors:** 


### Sun Stroke,

And stroke of lightning, as far as present light extends, cause death in the same manner; the blood is expanded and gases are liberated, both tending to distend the veins, which causes in the brain a species of appoplexy; this distension of the blood-vessels induces pressure on the brain, and consequently all loss of sense and feeling; the muscles are paralyzed, all motion ceases, and the functions of the body are all arrested.

Apply cold cloths or ice bags to the head, and mustard plaster to the neck, with something to act on the bowels as soon as possible. But something more speedy than this is an imperative necessity in most cases, or death will ensue in a few moments. Skillful and eminent physicians in this country, upon actual experiment, founded upon a true philosophy, have ascertained that speedy recovery takes place within an hour if the patient is bled from both arms in the old-fashioned way. From the largest distended vein, the blood may only flow by drops at the first second or two, but as it flows freer, the relief becomes almost miraculous, and speedy and complete. Dr. S. W. Butler, editor of the "Medical and Surgical Journal" of Philadelphia, not only approves of this mode of treatment but has practiced it successfully.

